# Maximal Voluntary Activation of the Elbow Flexors Is under Predicted by Transcranial Magnetic Stimulation Compared to Motor Point Stimulation Prior to and Following Muscle Fatigue

**DOI:** 10.3389/fphys.2017.00707

**Published:** 2017-09-20

**Authors:** Edward W. J. Cadigan, Brandon W. Collins, Devin T. G. Philpott, Garreth Kippenhuck, Mitchell Brenton, Duane C. Button

**Affiliations:** ^1^Human Neurophysiology Laboratory, School of Human Kinetics and Recreation, Memorial University of Newfoundland St. John's, NL, Canada; ^2^BioMedical Sciences, Faculty of Medicine, Memorial University of Newfoundland St. John's, NL, Canada

**Keywords:** interpolated twitch technique, motor evoked potential, biceps brachii, triceps brachii, fatigue, isometric contractions

## Abstract

Transcranial magnetic (TMS) and motor point stimulation have been used to determine voluntary activation (VA). However, very few studies have directly compared the two stimulation techniques for assessing VA of the elbow flexors. The purpose of this study was to compare TMS and motor point stimulation for assessing VA in non-fatigued and fatigued elbow flexors. Participants performed a fatigue protocol that included twelve, 15 s isometric elbow flexor contractions. Participants completed a set of isometric elbow flexion contractions at 100, 75, 50, and 25% of maximum voluntary contraction (MVC) prior to and following fatigue contractions 3, 6, 9, and 12 and 5 and 10 min post-fatigue. Force and EMG of the bicep and triceps brachii were measured for each contraction. Force responses to TMS and motor point stimulation and EMG responses to TMS (motor evoked potentials, MEPs) and Erb's point stimulation (maximal M-waves, M_max_) were also recorded. VA was estimated using the equation: *VA%* = (1−*SITforce*/*PTforce*) × 100. The resting twitch was measured directly for motor point stimulation and estimated for both motor point stimulation and TMS by extrapolation of the linear regression between the superimposed twitch force and voluntary force. MVC force, potentiated twitch force and VA significantly (*p* < 0.05) decreased throughout the elbow flexor fatigue protocol and partially recovered 10 min post fatigue. VA was significantly (*p* < 0.05) underestimated when using TMS compared to motor point stimulation in non-fatigued and fatigued elbow flexors. Motor point stimulation compared to TMS superimposed twitch forces were significantly (*p* < 0.05) higher at 50% MVC but similar at 75 and 100% MVC. The linear relationship between TMS superimposed twitch force and voluntary force significantly (*p* < 0.05) decreased with fatigue. There was no change in triceps/biceps electromyography, biceps/triceps MEP amplitudes, or bicep MEP amplitudes throughout the fatigue protocol at 100% MVC. In conclusion, motor point stimulation as opposed to TMS led to a higher estimation of VA in non-fatigued and fatigued elbow flexors. The decreased linear relationship between TMS superimposed twitch force and voluntary force led to an underestimation of the estimated resting twitch force and thus, a reduced VA.

## Introduction

Voluntary activation (VA) is the level of neural drive from the central nervous system to produce a given force output from a muscle. Examining how VA is estimated is important for quantifying the presence of central fatigue in clinical populations and for multiple research purposes (Taylor et al., 1996; Newham and Hsiao, 2001; Todd et al., 2003, 2005; Prasartwuth et al., 2005; Hunter et al., 2008; Cahill et al., 2011; Pearcey et al., 2015, 2016). The Interpolated Twitch Technique (ITT) was developed as a way to estimate central VA (Merton, 1954). The amplitude of an evoked superimposed twitch (SIT) force via an electrical stimulus to a nerve during a muscle contraction was expressed as a percentage of the amplitude of an evoked twitch force following the contraction when the muscle was at rest and in a potentiated state (Belanger and McComas, 1981). Transcranial magnetic stimulation (TMS) has also been used to estimate VA (Gandevia et al., 1996; Todd et al., 2003, 2004; Sidhu et al., 2009a). Due to recruitment of very few motor units (Hess et al., 1987; Ugawa et al., 1995; Di Lazzaro et al., 1998), TMS evokes a low amplitude potentiated twitch (PT) at rest following a muscle contraction. Therefore, a method was developed by linearly extrapolating the regression between TMS evoked SIT forces of submaximal voluntary contractions and MVCs to estimate a TMS-induced resting PT (Todd et al., 2003). Central and, in part, cortical VA can then be estimated by expressing a TMS evoked twitch force during a contraction as a percentage of the estimated PT at rest (Todd et al., 2003, 2004; Goodall et al., 2009; Sidhu et al., 2009a; Hunter et al., 2016).

Studies have directly compared the estimation of VA via nerve stimulation to TMS and have yielded comparable results (Todd et al., 2003; Sidhu et al., 2009a,b; Bachasson et al., 2016). However, they also differ for several reasons. TMS may activate motor units of synergist muscles leading to greater joint torque, whereas nerve stimulation may fail to activate all motor units, thus leading to differences in SIT force. VA and force forms a curvilinear relationship from 0 to 100% MVC with nerve stimulation (Todd et al., 2003; Shield and Zhou, 2004) as opposed to a linear relationship from 50 to 100% MVC with TMS (Todd et al., 2003, 2004; Lee et al., 2008; Goodall et al., 2009; Bachasson et al., 2016). There is a non-equivalent TMS estimated twitch force and the nerve stimulation PT force amplitudes for both the elbow flexors (Todd et al., 2003, 2004, 2005; Smith et al., 2007; Kennedy et al., 2013), and knee extensors (Goodall et al., 2009, 2012, 2014; Sidhu et al., 2009b; Klass et al., 2012). Fatigue in the central and peripheral nervous systems (Enoka and Stuart, 1992; Gandevia, 2001; Kent et al., 2016) reduces force production, alters SIT forces during submaximal voluntary contractions and MVCs and decreases estimated or resting PT forces (Todd et al., 2003; Goodall et al., 2009; Kennedy et al., 2013; Keller-Ross et al., 2014; Pearcey et al., 2015, 2016). The linear relationship between voluntary force and TMS evoked SIT force also decreases with fatigue resulting in an altered estimated resting twitch force and subsequently an over or underestimation of VA (Hunter et al., 2006, 2008; Kennedy et al., 2013; Yoon et al., 2013). There are also other technical challenges as described elsewhere (Shield and Zhou, 2004; Todd et al., 2016) with nerve stimulation and TMS for optimizing the estimation of VA.

There are few studies directly comparing nerve stimulation to TMS for estimating VA of the elbow flexors prior to, throughout and following bouts of fatiguing contractions especially in the elbow flexors. This is especially important because TMS and nerve stimulation are the two most commonly used stimulation techniques for indirectly measuring VA in the elbow flexors and whether or not one stimulation type is superior to another, especially during fatigue, warrants further investigation. Therefore, the purpose of this study was to compare nerve stimulation to TMS for estimating elbow flexor VA and how this estimation changes throughout and following a series of fatiguing MVCs. We hypothesized that nerve stimulation and TMS would estimate VA: (1) similarly in non-fatigued elbow flexors and (2) differently during and following fatigue.

## Materials and methods

### Participants

Based on prior research (Taylor et al., 2000), a statistical power analysis determined that six participants were necessary to achieve an alpha of 0.05 with a power of 0.8. Ten resistance-trained males (183.1 ± 5.9 cm, 92.5 ± 12.1 kg, 25.5 ± 4.9 years) from the university population were recruited for the study. Participants were considered resistance trained because they had all trained on average ≥3 sessions a week for ~an hour each session for at least 1 year. Participants were verbally informed of the procedures to be used during testing, and all gave informed written consent and completed a magnetic stimulation safety checklist to screen for potential contraindications with magnetic stimulation procedures (Rossi et al., 2009). The study was approved by the Memorial University of Newfoundland Interdisciplinary Committee on Ethics in Human Research (#20161806-HK) and was in accordance with the Tri-Council guidelines in Canada with full disclosure of potential risks to participants.

### Elbow flexor force

**Figure 1:**
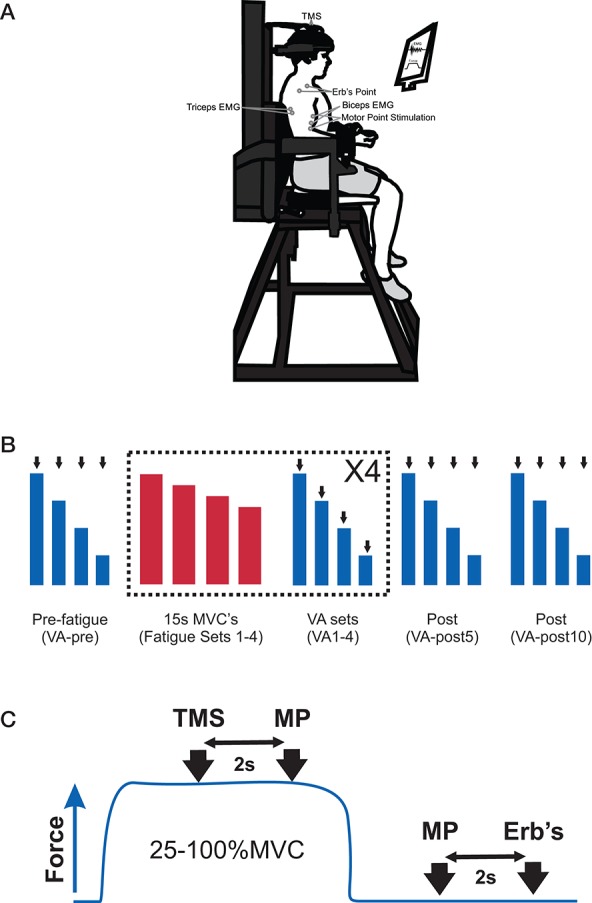
**(A)** Picture of the experimental set-up to measure elbow flexors submaximal and maximum voluntary contractions (MVC), voluntary activation (VA), and electromyography (EMG) and placement of EMG electrodes, transcranial magnetic stimulation (TMS), motor point stimulation and Erb's point stimulation. **(B)** Participants' performed an experimental protocol that consisted of a set of pre-fatigue voluntary contractions (100, 75, 50, 25% MVC, VA-pre), 4 sets of fatiguing contractions and 4 sets of voluntary contractions (100, 75, 50, 25% MVC, VA 1–4) and a set of voluntary contractions (100, 75, 50, 25% MVC) at 5- and 10-min post-fatigue (VA-post 5 and 10). A set of VA contractions was always performed following a set of fatiguing contractions. The VA contraction sets were performed in order to derive an estimated potentiated resting twitch (see Materials and Methods for details) to estimate VA. The black arrows indicate that the participant received several stimuli. The blue boxes indicate VA set contractions and the red bars represent fatiguing set contractions. **(C)** For each contraction (25–100% MVC) in the VA set, participants received TMS and motor point stimulation (at 2 and 4 s, respectively) and motor point stimulation and Erb's point stimulation (at 2 and 3 s, respectively) following the contraction when the elbow flexors were at rest. The blue trace represents one contraction in the VA set.

Participants were seated in a custom-built chair (Technical Services, Memorial University of Newfoundland) in an upright position, with hips and knees flexed at 90°, and head strapped in place to minimize movement (see Figure 1A). Both arms were slightly abducted with elbows resting on padded support at an angle of 90°. The forearms were held horizontal in a position midway between neutral and supination, and placed in a custom-made orthosis that was connected to a load cell (Omegadyne Inc., Sunbury, Ohio, USA). The load cell detected force output, which was amplified (x1,000) (CED 1902, Cambridge Electronic Design Ltd., Cambridge, UK) and displayed on a computer screen. Data was sampled at 2,000 Hz. Participants were asked to maintain the upright position during contractions. Verbal encouragement and visual feedback were given to all participants during all contractions.

### Electromyography

Electromyography (EMG) activity was recorded from the biceps brachii and lateral head of the triceps brachii muscles on the dominant arm using surface EMG recording electrodes (MediTrace Ag-AgCl pellet electrodes, disc shaped and 10 mm in diameter, Graphic Controls Ltd., Buffalo, N.Y., USA). Electrodes were placed length wise over the middle of the muscle belly with an interelectrode (center-to-center) distance of 2 cm and in accordance with SENIAM recommendations (Hermens et al., 2000). A ground electrode was placed over the lateral epicondyle of the dominant knee. Skin preparation for all recording electrodes included shaving to remove excess hair and cleaning with an isopropyl alcohol swab to removal of dry epithelial cells. An interelectrode impedance of <5 kΩ was obtained via a standard multimeter prior to recording to ensure an adequate signal-to-noise ratio. EMG signals were amplified (×1,000) (CED 1902) and filtered using a 3-pole Butterworth filter with cutoff frequencies of 10–1,000 Hz. All signals were analog-digitally converted at a sampling rate of 5 kHz using a CED 1401 (Cambridge Electronic Design Ltd., Cambridge, UK) interface.

### Stimulation conditions

#### Brachial plexus (Erb's point) stimulation

Stimulation of the brachial plexus (i.e., Erb's point) was used to induce a maximal compound muscle action potential (M_max_). Erb's point was electrically stimulated via a cathode on the skin in the supraclavicular fossa and an anode on the acromion process. Current pulses were delivered as a singlet (200 μs duration, 100–250 mA) via a constant current stimulator (DS7AH, Digitimer Ltd., Welwyn Garden City, UK). The electrical current was gradually increased until M_max_ of the biceps brachii was observed. To ensure maximal stimulation throughout the experiment, a supramaximal stimulation current (i.e., 130% greater than that required to elicit M_max_) was used (Todd et al., 2003; Goodall et al., 2012; Aboodarda et al., 2015; Pageaux et al., 2015).

#### Motor point stimulation

Electrical stimulation was delivered via a cathode placed on the skin over the biceps motor point and an anode on the brachii distal tendon (Smith et al., 2007; Khan et al., 2011; Monks et al., 2016; Pearcey et al., 2016). Current pulses were delivered as a doublet (10 ms apart, 100 μs duration, 100–225 mA) via a constant current stimulator (DS7AH, Digitimer Ltd., Welwyn Garden City, UK). The electrical current was gradually increased until there was no longer an increase in the twitch force of the elbow flexors. A supramaximal stimulation current (i.e., 130% greater than that required to elicit a maximum twitch force) was used for the remainder of the experiment (Allen et al., 1998).

#### Transcranial magnetic stimulation (TMS)

TMS (transcranial magnetic stimulator; Magstim 200, maximal output 2.0 Tesla) was delivered through a circular coil (13 cm outside diameter) placed directly over the vertex (Todd et al., 2003, 2004; McNeil et al., 2011; Forman et al., 2014; Pearcey et al., 2014; Philpott et al., 2015). The vertex was located by marking the measured halfway points between the nasion and inion and tragus to tragus. The intersection of these two points was defined as the vertex. Electrical currents flowed in an anticlockwise direction through the circular coil. The coil was placed horizontally over the vertex so that the direction of the current flow in the coil preferentially activated the right or left motor cortex (A side up for right side, B side up for left) for the activation of the dominant elbow flexors. Stimulation intensity (50–90% MSO) was adjusted to elicit a large MEP in the biceps brachii (>50% of M_max_) and a small MEP in the triceps brachii (<22% of the raw biceps brachii MEP amplitude) in the triceps brachii during elbow flexor MVCs (Todd et al., 2016). This stimulation intensity was used for the remainder of the experiment.

### Experimental set-up

Participants completed a familiarization and an experimental session, which was separated by at least 48 h. During the familiarization session participants received the stimulation conditions (TMS, brachial plexus, and motor point stimulation) to ensure they were comfortable with each stimulation. Participants then performed maximal elbow flexor isometric contractions, with 2 min in between each contraction, until they were able to reach peak force within 2 s. Next, they practiced elbow flexor contractions at the various percentages of the highest MVC (25, 50, 75%). Finally, the participants completed three fatiguing contractions (15 s long) similar to those to be performed in the experimental session.

During the experimental session participants were prepped for the stimulation conditions and EMG. Next, maximal twitch force and M_max_ were obtained at rest through motor and Erb's point stimulation, respectively. Participants then completed a series of brief (2–3 s) elbow flexor MVCs. During the MVCs the participants received TMS to determine the necessary intensity to elicit a MEP amplitude that was >50% of the M_max_ which was measured from the biceps brachii during the resting twitches. Following each of the brief MVCs, motor point stimulation was administered once again in order to evoke a PT force and to ensure maximal potentiation (Kufel et al., 2002).

The participants then started the experimental protocol. The protocol consisted of two different types of elbow flexor contractions; contractions to determine VA and contractions to induce fatigue. All of the contractions to determine VA included a MVC followed by randomly performing 25, 50, and 75% of the MVC. Each contraction was ~5 s in duration. The submaximal contractions (25, 50, and 75% of MVC) were always made relative to the 100% MVC in each set. All forces were displayed on a computer screen, which enabled the participants to match the target force. During each maximal and submaximal contraction participants received TMS and motor point stimulation at 2 and 4 s, respectively. Two and three seconds following the completion of the 5 s contraction, when the elbow flexors were at rest, participants received another motor point stimulation and an Erb's point stimulation, respectively. The fatigue contractions consisted of 3, 15 s sustained elbow flexor MVCs with 5 s rest between each sustained MVC. Although participants force declined during each 15 s MVC due to fatigue, they were verbally encouraged to maximally contract the elbow flexors throughout the entire 15 s contraction.

Initially, participants performed a set of VA elbow flexor contractions. Following the VA contractions, they started the fatigue contractions. After the completion of 3 fatigue contractions they immediately completed another VA set. This process was repeated 3 times. Additional sets of VA contractions were performed at 5 and 10 min post-fatigue contractions. In total participants completed 4 sets of fatigue contractions (12 sustained MVCs) and 7 sets of the VA contractions at pre-fatigue (VA-pre), following fatigue sets 1, 2, 3, and 4 (VA 1–4) and at 5- and 10-min post-fatigue (VA-post 5 and 10; see Figure 1 for experimental set-up).

### Data analysis

The Interpolated Twitch Technique (ITT) was utilized as a measure of the central nervous system's ability to fully activate the contracting muscle (Shield and Zhou, 2004). VA was calculated by comparing the amplitude of the SIT force with the actual or predicted PT force with the following equation: *VA%* = (1−*SITforce*/*PTforce*) × 100 and was quantified by measurement of the elbow flexor force responses to single pulse motor cortical stimulation and to double pulse motor point stimulation during 50, 75, and 100% MVC. The predicted resting PT force for each participant was derived from extrapolating the linear regression (*r*^2^ value) between the SIT forces upon the voluntary forces over the force ranges: 50, 75, and 100% MVC. These force ranges were chosen because they gave the best *r*^2^ values for TMS predicted twitch force (data not shown) for TMS (Todd et al., 2004; Goodall et al., 2009) and motor point stimulation and will be referred to as TMS predicted and motor point predicted hereafter. The **y**-intercept was taken as the estimated amplitude of the resting PT force. Each set of contractions provided a resting estimated PT force. Furthermore, VA was also quantified by measurement of the elbow flexor force responses to motor point stimulation during 100% MVC and divided by the resting PT force following the MVC (i.e., not using a predicted PT force and referred to as motor point actual hereafter) and a linear regression (*r*^2^ values) between the SIT forces upon the voluntary forces over the force ranges (25–100%) and the actual potential twitch (i.e., 0% MVC) and 25–100%. The amplitude of motor point actual PT force was also measured to assess muscle fatigue. The maximal force of the elbow flexors was quantified as the average value over a 500 ms interval that was centered about the peak of the MVC. The biceps and triceps brachii EMG activity was determined as the root mean square (RMS) value over a 500 ms interval about the same interval of the MVC force measurement. Triceps EMG was also expressed as a percentage of biceps EMG during each elbow flexor MVC.

The amplitudes and areas of MEP and M_max_ of the biceps and triceps brachii evoked by TMS and Erb's point stimulation, respectively, were measured between cursors placed at the beginning and end of the evoked potentials for each set of contractions. Triceps MEP amplitude was also expressed as a percentage of bicep MEP amplitude during each MVC. Because amplitude and area showed similar changes, only amplitude data were reported. All data were measured offline using Signal 4.0 software (Cambridge Electronic Design Ltd., Cambridge, UK).

### Statistical analysis

Statistical analyses were computed using SPSS software (SPSS 22.0, IBM Corporation, Armonk, New York, USA). Assumptions of sphericity (Mauchley test) and normality (Shapiro–Wilk test) were tested for all dependent variables. If the assumption of sphericity was violated, the corrected value for non-sphericity with Greenhouse-Geisser epsilon was reported. A one-way ANOVA with repeated measures (time; VA-pre, VA 1–4, and VA-post 5 and 10-min) was performed on MVC force, EMG, and PT force. A two-way ANOVA (3 × 7) with repeated measures (stimulation type; motor point actual, motor point predicted and TMS predicted × time) was performed on VA, actual and predicted twitch forces and r^2^ values. A two-way ANOVA (2 × 7) with repeated measures (stimulation type × time) was performed on super imposed twitch force. A Bonferonni *Post-hoc* test was performed to test for significant differences between interactions. *F*-ratios were considered statistically significant at the *p* < 0.05 levels. Cohen's *d* effects sizes (ES) (Cohen, 1988) were also calculated to determine the magnitude of the differences between interventions and time. The following criteria were used: ES < 0.2 “trivial”; ES = 0.2–0.49 “small”; ES = 0.5–0.79 “medium”; and ES > 0.8 “large.” Percentage changes and absolute values (mean ± *SD*) are reported in text and absolute values only (mean ± SE) are reported in **Figures 3**–**5**.

## Results

### MVC force and EMG

**Figure 2:**
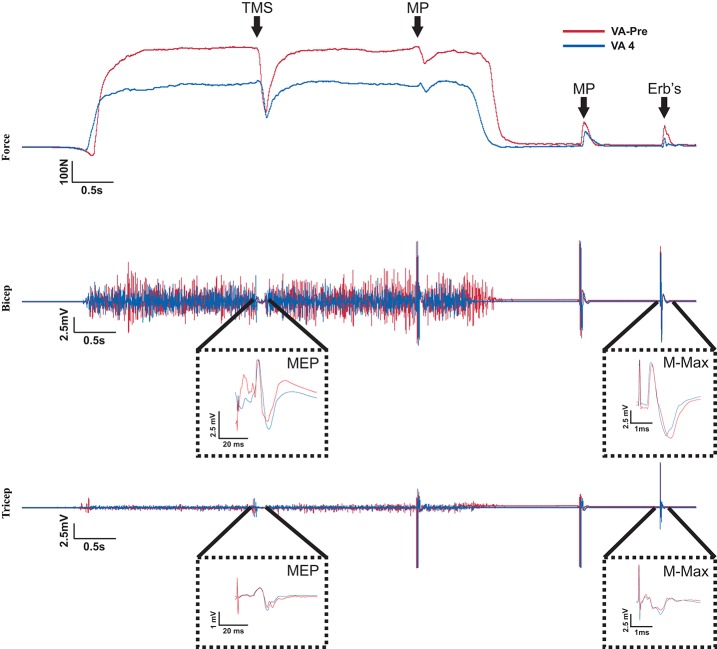
Raw Data recorded from one participant. The red and blue traces represent measures taken from 100% MVC of the elbow flexors in the VA-pre set and VA 4 set, respectively. Top of the figure shows the MVC forces and when the stimulation occurred during the MVCs. It also shows the stimulus evoked superimposed twitch forces during the MVCs and the stimulus evoked potentiated twitch forces following the MVCs. Notice the reduction in MVC and potentiated twitch forces and the increased superimposed twitch forces in VA 4 compared to VA-pre. The middle and bottom portions of the figure show the biceps and triceps brachii EMG recorded during the MVCs traces from the top of the figure. A MEP and M_*max*_ occurred in response to TMS and Erb's point stimulation, respectively for both the biceps and triceps brachii and are amplified for clarity. Notice the decrease in EMG during the VA-4 MVC compare to the VA-pre MVC. Also, there was no change in the MEP or M_max_ response.

**Figure 3:**
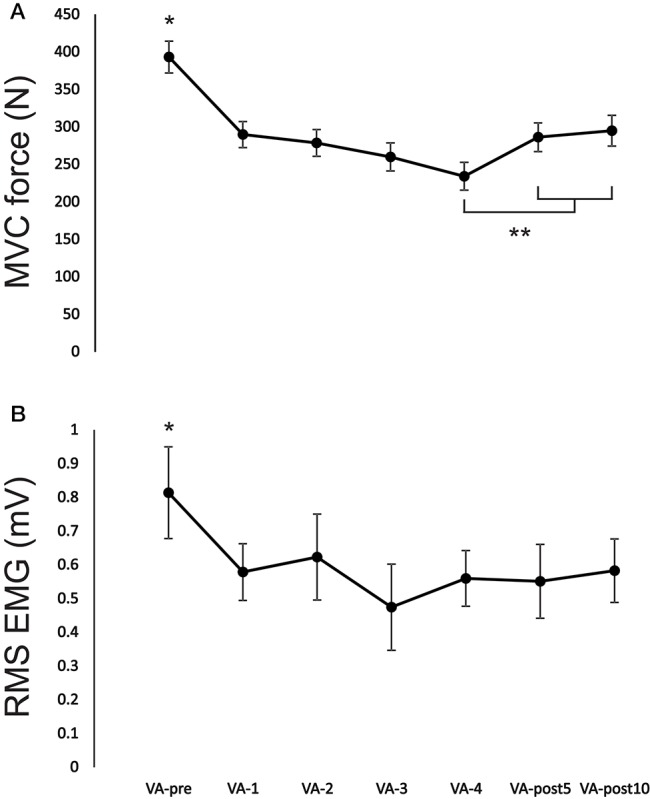
Change in **(A)** MVC force and **(B)** EMG during recorded during the MVC for each VA set from VA-pre to VA-post10. ^*^Indicates a significant difference (*p* < 0.01) between VA-pre and all other time points and ^**^indicates a significant difference (*p* < 0.05) from VA-4. Each data point represents the group mean ± SE.

There was a significant [*F*_(6, 54)_ = 22.47, *p* < 0.001; *F*_(6, 54)_ = 2.57, *p* = 0.03; *F*_(6, 54)_ = 18.77, *p* < 0.001] main effect for time on MVC force, EMG and PT force, respectively. MVC force significantly (*p* < 0.01, ES = 2.7) decreased from VA-pre to VA-4 by 40.5 ± 9.1% (Figures 2, 3A). MVC force remained significantly (*p* < 0.01, ES = 1.7 and *p* < 0.01, ES = 1.5) depressed by 27.2 ± 9.3 and 24.9 ± 11.3% at VA-post5 and VA-post10, respectively compared to pre-fatigue. However, MVC force significantly (*p* < 0.01, ES = 0.9 and *p* < 0.01, ES = 1.0) increased by 22.2 ± 18.4 and 25.9 ± 19.1% at VA-post5 and VA-post10, respectively compared to VA-4. EMG significantly (range: *p* < 0.01–*p* = 0.03, ES = 0.8–1.4) decreased (range: 24.9 ± 20.1–44.6 ± 31.1%) from VA-pre compared to all other time points (Figure 3B).

### Voluntary activation

**Figure 4:**
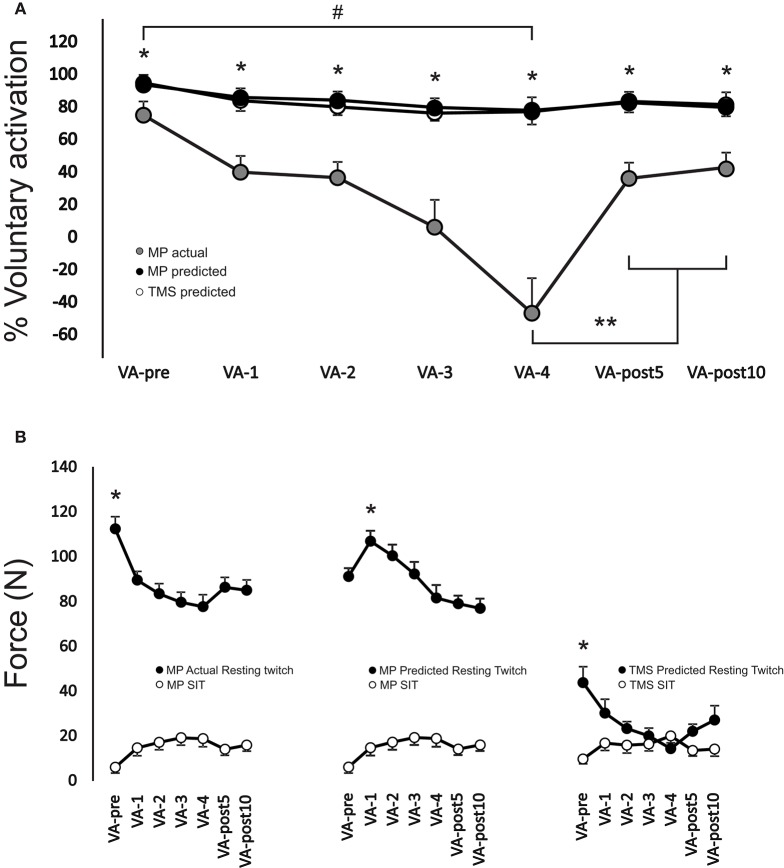
**(A)** Change in voluntary activation during the MVC for each VA set from VA-pre to VA-post10. ^*^Indicates a significant difference (*p* < 0.01) between TMS predicted compared to motor point actual and motor point predicted, ^**^indicates a significant difference (*p* < 0.05) from VA-4 for TMS predicted only and # represents a significant main effect (*p* < 0.05) for time for VA-pre compared to VA-4. **(B)** Change in motor point actual, motor point predicted and TMS predicted resting potentiated twitch forces (black filled circles) and motor point and TMS evoked superimposed twitch forces (white filled circles) at 100% MVC. ^*^Indicates a significant difference (*p* < 0.01) between VA-pre and all other time points for both motor point actual and TMS predicted and VA-1 and all other time points for motor point predicted resting potentiated twitch forces. All time points for motor point actual and motor point predicted resting potentiated twitch forces were significantly different (*p* < 0.01) than those of TMS predicted resting potentiated twitch forces (not symbols shown to denote difference). Each data point **(A,B)** represents the group mean ± SE.

There was a significant interaction [*F*_(12, 108)_ = 10.54, *p* < 0.001] between stimulation type and time for VA (Figure 4A). VA when using motor point actual or motor point predicted twitch forces were significantly (*p* < 0.01 for all time points, ES = 1.6–3.1) higher at each time point (range: 20.9 ± 11.9–136.1 ± 39.6%) compared to using TMS predicted twitch force. VA significantly (*p* < 0.01, ES = 1.6; *p* < 0.01, ES = 1.8; *p* < 0.01, ES = 1.8) decreased from VA-pre to VA-4 by 17.7 ± 9.0, 20.4 ± 8.4, and −75.2 ± 99.2% when calculated by using the motor point actual, motor point predicted, and TMS predicted twitch forces, respectively. VA remained significantly depressed by 16.7 ± 5.7 and 15.7 ± 6.2 (*p* < 0.01, ES = 1.1 and *p* < 0.01, ES = 1.3) when using motor point actual, by 19.4 ± 5.7 and 18.4 ± 6.6% (*p* < 0.01, ES = 1.2 and *p* < 0.01, ES = 1.4) when using motor point predicted and by 51.3 ± 44.5 and 58.6 ± 28.6% (*p* < 0.01, ES = 1.7 and *p* < 0.01, ES = 1.5) when using TMS predicted twitch forces at VA-post5 and VA-post10, respectively compared to VA-pre. However, VA significantly (*p* < 0.05, ES = 3.3 and *p* < 0.05, ES = 4.0) increased by 176.9 ± 46.6 and 191.3 ± 44.3% at VA-post5 and VA-post10, respectively compared to VA-pre, when using TMS predicted twitch forces.

There was a significant interaction [*F*_(12, 108)_ = 9.33, *p* < 0.001] between stimulation type and time for PT force (Figure 4B). Motor point actual and motor point predicted resting potentiated twitch forces were significantly (*p* < 0.01 for all time points, ES = 2.7–5.2) higher at each time point (range: 50.1 ± 23.6–79.6 ± 13.1%) compared to TMS predicted resting potentiated twitch force. Motor point actual was significantly (*p* < 0.05, ES = 1.7) higher by 19% at VA-pre compared to motor point predicted resting potentiated twitch force at VA-pre. Motor point actual and TMS predicted resting potentiated twitch forces significantly (*p* = 0.001–*p* = 0.003, ES = 1.8–2.2 and *p* < 0.01–*p* = 0.034, ES = 0.7–3.6) decreased from VA-pre compared to all other time points, respectively by 19.9 ± 8.5–30.5 ± 13.3 and 27.1 ± 25.1–40.1 ± 34.1%. Motor point predicted resting potentiated twitch forces significantly (*p* < 0.01, ES = 1.3) increased by 17.7 ± 10.2% from VA-pre to VA-1 and significantly (*p* < 0.01, ES = 1.1 and *p* < 0.01, ES = 1.1) decreased by 14.5 ± 14.7 and 15.4 ± 12.1% from VA-1 to VA-post5 and VA-post10, respectively.

**Figure 5:**
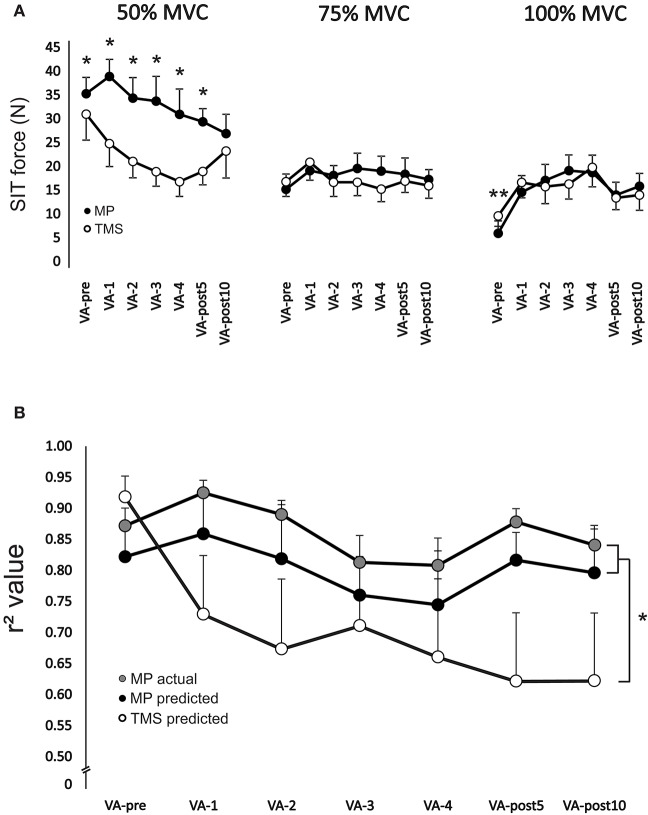
**(A)** Change in motor point stimulation and TMS evoked superimposed twitch forces during 50% (left), 75% (middle), and 100% (right) MVCs. ^*^Indicates a significant difference (*p* < 0.05) between stimulation type and ^**^indicates a significant difference (*p* < 0.05) between VA-pre and all other time points. **(B)** Change in *r*^2^ values for predicting resting potentiated twitch force from motor point stimulation and TMS for each set of voluntary contractions from VA-pre to VA-post10. *R*^2^ values were calculated as the relationship between motor point and TMS evoked superimposed twitch forces at 50, 75, and 100% MVC and 50, 75, and 100% MVC forces. *R*^2^ values were also calculated as the relationship between motor point evoked superimposed twitch forces at 0 (i.e., following the 100% MVC at rest), 25, 50, 75, and 100% MVC and 0, 25, 50, 75, and 100% MVC forces. ^*^Indicates a significant main effect (*p* < 0.01) for stimulation type. Each data point **(A,B)** represents the group mean ± SE.

There was a significant [*F*_(6, 54)_ = 8.64, *p* < 0.001] main effect for time on SIT force at 100% MVC. SIT force significantly (*p* < 0.01 for all time points, ES = 0.7–1.3) increased (range: 159.4 ± 120.1–253.9 ± 210.1%) from VA-pre compared to all other time points (Figure 5A). There was a significant interaction [*F*_(6, 36)_ = 6.0, *p* < 0.001] between stimulation type and time for SIT force at 50% MVC. TMS SIT force was 30.2 ± 20.2–35.7 ± 12.9% lower (*p* < 0.05 for all time points, ES = 1.0–1.6) than motor point stimulation from VA-1 to VA-post10, respectively (Figure 5A).

There was a significant main effect [*F*_(1, 8)_ = 5.9, *p* < 0.05] for stimulation type on *r*^2^ values. Overall, *r*^2^ values were significantly (*p* < 0.05 and *p* < 0.05) lower by 19.9 ± 5.1 and 14.2 ± 5.5% for TMS predicted than motor point actual and predicted, respectively (Figure 5B).

### Biceps and triceps EMG and MEP and M_max_ amplitudes

There was no significant [*F*_(6, 54)_ = 0.83, *p* = 0.53; *F*_(6, 54)_ = 2.66, *p* = 0.08; *F*_(6, 54)_ = 3.82, *p* = 0.06; *F*_(6, 54)_ = 2.55, *p* = 0.08] main effect for time on 100% elbow flexor MVC triceps/biceps EMG, biceps MEP, and M_max_ amplitudes, and triceps/biceps MEP amplitude, respectively. Triceps EMG ranged from 18.9 ± 12.1 to 22.2 ± 14.1% of biceps EMG from VA-pre to VA-post10. Biceps MEP and M_max_ amplitudes ranged from 6.7 ± 4.5 to 7.8 ± 4.1 and 13.5 ± 6.2 to 13.9 ± 5.8 mV, respectively from VA-pre to VA-post10. Triceps MEP ranged from 15.1 ± 8.2 to 21.4 ± 11.2% of biceps MEP from VA-pre to VA-post10.

## Discussion

Overall elbow flexor fatigue was due to a combination of reduced output from the central and peripheral nervous systems. Interestingly, VA was substantially underestimated when using TMS compared to motor point stimulation in non-fatigued and fatigued elbow flexors. As elbow flexor fatigue developed, this underestimation became dispersed. The dispersed underestimation of VA could not be explained by a fatigue-induced increase of triceps brachii activation, but instead a reduced linearity between the TMS evoked SIT force and voluntary force of the elbow flexors. The decreased linearity subsequently yielded a reduced TMS, but not motor point stimulation, predicted resting twitch force leading to an underestimation of VA. The reduced linearity may be due to TMS evoked SIT force being much smaller than motor point stimulation evoked SIT forces at 50% MVC.

The elbow flexor fatigue protocol in the current study induced fatigue both centrally and peripherally. Participants could no longer voluntarily drive the muscle the same way as pre-fatigue. Following the fatiguing contractions, motor point stimulation evoked larger SIT forces during the MVCs than when the muscles were not fatigued indicating that the axons of the motoneurones were capable of increased output but that there was a reduction in central nervous system output to (i.e., at the corticomotoneuronal synapse; Gandevia et al., 1999) or within the motoneurone itself (i.e., decreased intrinsic excitability; Khan et al., 2012). During the same MVCs, the increased SIT force due to TMS indicates that the reduced output to the motoneurone was due, in part, to altered synaptic activity from the motor cortex (Taylor et al., 1996; Ranieri and Di Lazzaro, 2012). Because there was no change in the biceps brachii MEP amplitude, the corticospinal pathway probably never played a role in the reduced MVC force. Thus, the altered synaptic activity to the motoneurone may be upstream from this pathway. Mechanisms of fatigue-induced changes in central nervous system output have been reviewed elsewhere (Gandevia, 2001; Ranieri and Di Lazzaro, 2012; Kent et al., 2016). The reduction in PT force indicates that the reduction in MVC force was, in part, due to fatigue induced changes in the elbow flexor muscles. The reduction in PT illustrates that there were impairments to: (1) muscle excitation-contraction coupling, such as sarcoplasmic reticulum release, restoration of intracellular calcium and sensitivity of calcium to contractile protein interactions, (2) H+, (3) PCr breakdown, (4) muscle deoxygenation, and (5) others, which have all been reviewed in detail (Enoka and Stuart, 1992; Fitts, 1994; Allen et al., 2008; Kent et al., 2016).

The most interesting finding in the current study was the disperse differences in estimated VA via TMS compared to motor point stimulation, especially during the development of elbow flexor fatigue. A potential reason for these differences is antagonist co-activation. Because cortical stimulation is not focal, there may be an activation of corticospinal cells that project to various muscles including the antagonist. Activation of the antagonist during an agonist contraction would reduce the size of the SIT force and subsequently result in an over- or underestimation of VA (Todd et al., 2016). In the current study, at all contraction intensities (data only shown for 100% MVC) and throughout the development of fatigue the triceps/biceps MEP and EMG ratios were ~20% or less. Thus, increased antagonist activation could not explain the disperse VA differences between TMS and motor point stimulation or the decrease in VA via TMS from VA-pre to VA-4.

The main reason for the disperse differences in VA as the participants fatigued was poor linear regression. When linear regression between motor point evoked SIT force and voluntary force was made the average *r*-values at all times points were high for the elbow flexors with and without fatigue. For TMS the linear regression average *r*-value was high only for the elbow flexors in a non-fatigued state. It has been shown that non-linearity of the regression between TMS evoked SIT force and voluntary force occurs more often with fatigued compared to non-fatigue muscle (Hunter et al., 2006, 2008; Girard et al., 2013; Kennedy et al., 2013; Keller-Ross et al., 2014). Based on the current findings, the difference in the linear regression between TMS and motor point stimulation was due to the 50% but not 75 or 100% MVCs. The evoked SIT forces were similar for both TMS and motor point stimulation at 75 and 100% MVC but much smaller for TMS at 50% MVC. These differences lead to an underestimation of resting twitch force for TMS. In fact, because of these differences in linearity, the TMS predicted resting twitch force became so underestimated that by VA-4 the SIT was larger than the predicted resting twitch, and thus a negative VA occurred. As the SIT force at 50% MVC started to recover post-fatigue there was an increase in the estimated resting twitch force and VA became positive again.

There were several methodological considerations for this study. Typically, the TMS predicted resting twitch force is larger in the elbow flexors compared to the resting potentiated twitch force evoked by motor point stimulation (Todd et al., 2003, 2004; Kennedy et al., 2013), which is opposite to what happened in the current study. The differences between TMS and motor point predicted VA compared to other studies (Todd et al., 2003, 2004; Kennedy et al., 2013) may have occurred for several reasons. First, in the aforementioned studies a single motor point stimulus was delivered, whereas a double stimulus was used here. A double rather than a single stimulus was used to evoke twitch forces because it has been shown to produce a higher signal to noise ratio (Behm et al., 1996). The double stimulus at the motor point recruited the elbow flexor muscle fibers differently than TMS at 50% MVC especially during the fatiguing contractions. Second, we recruited chronically strength-trained participants, whereas other studies (Todd et al., 2003, 2004; Kennedy et al., 2013) did not. Although, strength-training alters various sites in the central nervous system (Carroll et al., 2011) it does not appear to affect VA of upper limb muscles (Herbert et al., 1998; Lee et al., 2009). Lastly, in the current study, the elbow joint was flexed to 90° and the shoulder at 0° with the forearm parallel to the ground and supinated with the force at the wrist being upwards. In other studies, (Todd et al., 2003, 2004; Kennedy et al., 2013) the elbow and shoulder joints were flexed to 90° with the forearm vertical and supinated with the force at the wrist being backwards. Changes in forearm and shoulder positions alters CSE of the biceps brachii (Forman et al., 2016; Nuzzo et al., 2016) and potentially could affect VA of the elbow flexors. Todd et al. (2003) showed a high linear regression between TMS evoked SIT forces and voluntary force (50–100% MVC) in fatigued elbow flexors with the elbow and shoulder joints were flexed to 90°, which was opposite to the current results. However, to the best of our knowledge no studies to date have determined the combined effects of fatigue, shoulder position, stimulation type and training on elbow flexor VA.

## Conclusion

The estimation of VA or the level of neural drive from the central nervous system to produce force is important for quantifying the presence of central fatigue in various physiological conditions. Compared to motor point stimulation, VA of the elbow flexors was underestimated prior to and even more so during fatigue when using TMS. During fatigue the stimulus evoked SIT forces responded differently to TMS at submaximal compared to near maximal or maximal voluntary contractions leading to an underestimation of the resting twitch and subsequently underestimation of VA. TMS during voluntary contraction does have the advantage over motor point stimulation to indicate that a change in VA is, in part, cortex dependent. However, based on the current findings and the conditions in which VA was measured the use of TMS to estimate VA of the elbow flexors may not be an appropriate technique especially following fatigue. Overall motor point stimulation was the more appropriate technique for estimating VA of the elbow flexors.

## Authors contributions

EC, BC, DP, GK, MB, and DB contributed to the conception or design of the work, the acquisition, analysis, and interpretation of data for the work and final submission of the manuscript. All authors agree to be accountable for the work.

### Conflict of interest statement

The authors declare that the research was conducted in the absence of any commercial or financial relationships that could be construed as a potential conflict of interest.

## Abbreviations

VA: Voluntary Activation
CSE: corticospinal excitability
TMS: transcranial magnetic stimulation
MEP: motor evoked potential
MVC: maximum voluntary contraction
Mmax: maximal muscle compound action potential
EMG: electromyography
PT: potentiated twitch
ITT: interpolated twitch technique
SIT: superimposed twitch
RMS: root mean square.
